# The impact of helminth infection in fish farms on wild trout populations: host immune regulation of disease risk

**DOI:** 10.1017/S0031182025101029

**Published:** 2025-11

**Authors:** Richard C. Tinsley, Abigail M. Carey, Miguel Rubio-Godoy

**Affiliations:** 1 School of Biological Sciences, University of Bristol, Bristol, UK; 2 Red de Biología Evolutiva, Instituto de Ecología, A.C., Xalapa, Veracruz, Mexico

**Keywords:** *Discocotyle*, fish farms, immune regulation of parasites, infection spillover, Monogenea, *Oncorhynchus mykiss*, *Salmo trutta*

## Abstract

Intensive rearing of farmed fish may risk disease spillover into free-living populations. This study concerns the blood-feeding gill monogenean of salmonids, *Discocotyle sagitatta*, on the Isle of Man, UK. Heavy infections in 2 fish farms have led to severe disease with periodic mass mortality. Infection levels in rainbow trout (*Oncorhynchus mykiss*, overall *n* = 556) increased with age (i.e. years exposed): by year 3, prevalence was 100%, mean intensity *c.* 100 (maximum 1150) worms/host. Output from farms of many millions of parasite eggs/day has the potential for transmission to downstream populations of free-living trout. Infections of *Discocotyle sagittata* were recorded in 132 brown trout and 49 sea trout (*Salmo trutta*) at 9 sites in rivers associated with or independent of the farms. Its occurrence in all 5 rivers studied confirmed that it is endemic on the Isle of Man irrespective of the farms. Wild brown and sea trout in rivers local to the farms (Rivers Corrany and Neb) had similar burdens to fish from independent drainage systems (Rivers Laxey, Santon and Sulby), and all burdens were within the range reported for other free-living populations in the distribution of *D. sagittata*. Low worm burdens in brown trout persisted even where these occurred in farm ponds contiguous with heavily infected rainbow trout. It had seemed predictable that high worm burdens in the farms would increase infection in downstream wild fish, but no elevation was detectable. Instead, this and other studies indicate that brown trout develop protective immunity despite intensive re-infection from rainbow trout, preventing pathogenic disease.

## Graphical abstract

**Figure:**
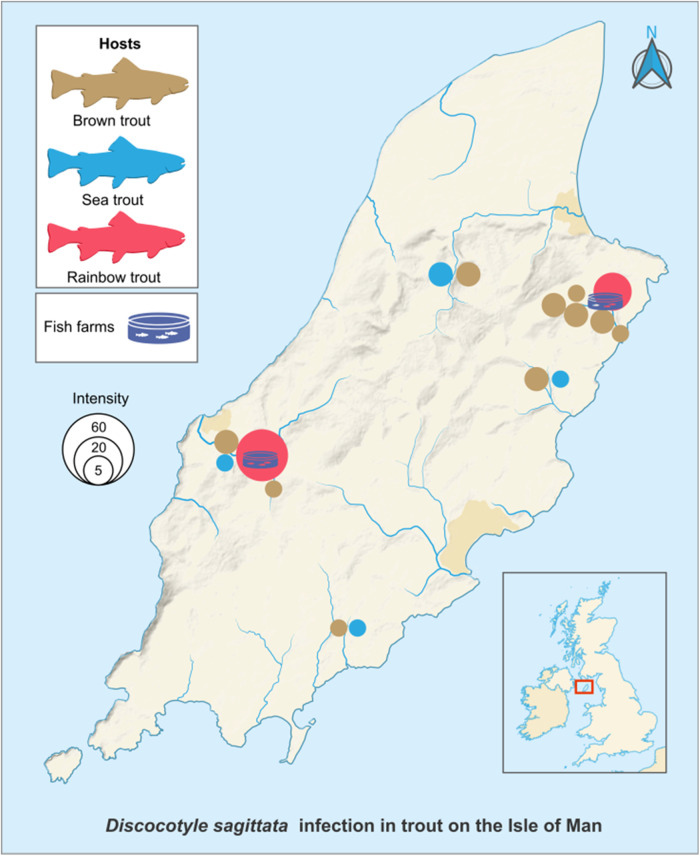

## Introduction

There is relatively extensive documentation demonstrating that environmental conditions created for intensive rearing of farmed fish species may have important implications for the build-up of pathogenic disease. High fish population density and stress may result in amplification of infections to levels seldom seen in natural ecosystems. Greatest current concerns focus on viruses, bacteria and parasitic copepods (e.g. Costello, 2009; Krkošek *et al.* 2011; Austin and Austin, 2016; Krkošek, 2017; Kibenge, 2019; Shea *et al*. 2020; Bouwmeester *et al*. 2021; Shinn *et al*. 2023; Timi and Buchmann, 2023). In addition, several helminth species, which are generally considered relatively harmless or mildly pathogenic in wild fish, have been shown to be the causative agents of serious disease outbreaks in cultured species (Woo and Buchmann, 2012; Soler-Jiménez *et al*. 2017). These include the Monogenea with simple, direct life cycles that are completed easily in confined conditions both in freshwater and marine aquaculture (Thoney and Hargis, 1991; Soler-Jiménez *et al*. 2017; García-Vásquez *et al*. 2021; Esposito *et al*. 2023; Shinn *et al*. 2023; Mladineo *et al*. 2024; Mo, 2024). *Gyrodactylus* and related genera are notorious for their polyembryonic reproduction, generating rapid population growth *in situ* on the surface of fish (Bakke *et al*. 2007). Other monogeneans reproduce by means of eggs which are released and develop to an infective stage off the host, but re-infection is promoted by densely aggregated fish populations in confined habitats.

Favourable conditions for the build-up of parasite infections in aquaculture create a risk of disease transfer to wild fish via discharges from farms (McGuigan and Sommerville, 1985; Rubio-Godoy and Tinsley, 2008a). The present study focused on 2 trout farms in the Isle of Man, UK, where levels of infection by the monogenean *Discocotyle sagittata* in non-native, introduced rainbow trout (*Oncorhynchus mykiss*) are unusually high (including 100s or even 1000s of worms/host, e.g. Tinsley *et al*. 2020). Reproductive rates are, by comparison with most parasites, relatively low: egg production is around 8 eggs/worm/day (e/w/d) at 13°C (Gannicott and Tinsley, 1998a; Tinsley RC, 2004); larvae develop to an infective stage in 21–30 days and then reach maturity and begin egg production after about 10 weeks post-infection (*p.i.*). Worm burdens are rarely >5 worms/host in free-living populations of native brown trout (*Salmo trutta*) in UK lakes and rivers (references below), but factors influencing transmission in the wild contrast strongly with those characteristic of densely confined, static populations of rainbow trout in farms.

*Discocotyle sagittata* has a wide north temperate distribution infecting several salmonid species (Williams and Jones, 1994; Nikulina and Polyaeva, 2020; Mo, 2023); in the United Kingdom, it is native to brown trout but parasite burdens are significantly higher in rainbow trout (introduced from North America to the United Kingdom) (Gannicott, 1997). Both host species can develop partial immunity to re-infection, but different infection levels for these hosts when raised in the same farm conditions suggest different immune capabilities (Gannicott, 1997; Rubio-Godoy and Tinsley, 2008a) and the greater resistance of brown trout has been confirmed by laboratory experimental infections (Rubio-Godoy and Tinsley, 2004a).

*Discocotyle sagittata* attaches to the hosts’ gills and feeds on blood. In the Isle of Man farms, burdens of several hundred worms/host may cause severe anaemia and mortality, particularly when coinciding with elevated water temperatures (≥20°C) and low water flows in summer (Rubio-Godoy and Tinsley, 2008a; Tinsley *et al*. 2020). Host death may result from a combination of increased blood removal by parasites, increased oxygen demand by hosts, reduced blood oxygen-carrying capacity, and depleted oxygen concentrations in the water.

In addition to effects on host pathology, mortality and economic loss, the very large parasite populations are responsible for massive output of eggs. These contribute both to continuing intense transmission within the farm and to dispersal with outflow of farm effluent potentially affecting free-living fish populations. This leads to the logical expectation that downstream wild trout would be exposed to a force of infection far greater than that typical in natural watercourses resulting in elevated parasite burdens and the possibility of spill-over of pathogenic disease from farms into the wider environment.

The aims of the present investigation were to review infections of *D. sagittata* in the Isle of Man trout farms, to survey parasite populations in wild brown trout and sea trout (*S. trutta*) in rivers connected with the trout farms, to compare these with infections in independent waterways without links to the farms, and to determine whether the unnaturally high and pathogenic levels of parasite infection in farmed rainbow trout are a significant disease risk to wild fish.

## Materials and methods

### Collection of farm fish

**Figure 1.**
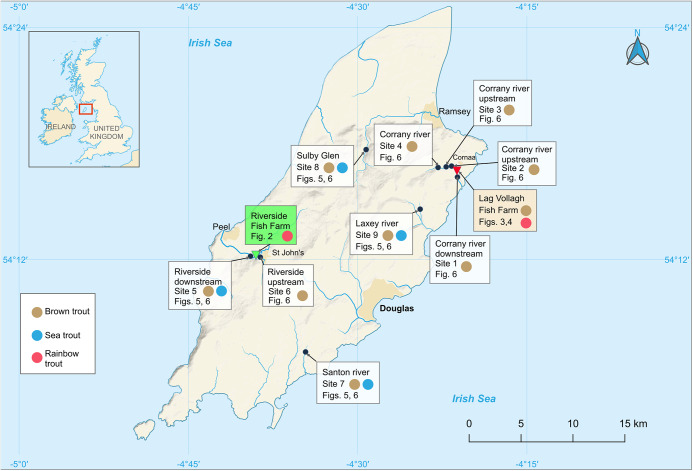
Map of the Isle of Man with insert showing the location of the island in the Irish Sea. Principal rivers (**∼**) indicate separate drainage systems with approx. positions of fieldwork collection sites (black dots). Site numbers correspond with the list in Materials and Methods (see text) with reference to the fish species collected (brown trout, sea trout, rainbow trout). The numbered figures cited give the infection data for the fieldwork samples. Triangles show locations of the fish farms: Riverside Farm, St. John’s, rearing rainbow trout, and Lag Vollagh Farm, Cornaa, rearing rainbow and brown trout. Sites upstream and downstream refer to location above and below Lag Vollagh farm (sites 1–4) and Riverside farm (sites 5–6) (see Materials and Methods).

Rainbow trout (*O. mykiss*) and brown trout (*S. trutta*) were sampled at 2 trout farms on the Isle of Man between November 1993 and December 1996. Additional studies were made in many subsequent years including the early 2000s (Rubio-Godoy and Tinsley, 2008a,b) up to 2012 (Tinsley *et al*. 2020). Some of these later published observations are incorporated into this account. Lag Vollagh is a government-funded farm at Cornaa on the north-east coast of the island, with inlets from the Corrany River (Figure 1). Both trout species are produced at the hatchery to restock local rivers and fishing reservoirs. Riverside trout farm, St. John’s, is a commercial farm near the mid-west coast of the island with a link to the River Neb (Figure 1). The rainbow trout produced are used to restock a small private fishing lake on site or sold for human consumption. The farms have similar Danish design with a series of ponds containing separate populations of fish connected by water flow from the pond above in the sequence and an outlet into the pond below. There are 9 ponds at Riverside and 10 at Lag Vollagh with typically 5–6000 fish per pond. The uppermost ponds at both farms receive inlets from the adjacent river supplying the whole sequence and there are additional inlets into the separate ponds supplementing the water flow from the pond above. The outflow from the system returns to the river downstream of the final pond (via a lake at Riverside). Thus, the pond populations of fish are separate from each other but they are united by interconnected water supplies which carry effluent (with parasite eggs and oncomiracidia) from pond to pond. During low river flow in dry summers, water may be pumped from the lowermost ponds up to the start of the sequence to improve flow rates through the system.

### Collection of wild fish

Brown and sea trout (*S. trutta*) were sampled in widely separated rivers on the Isle of Man. Six sites were local to Lag Vollagh and Riverside farms, situated both upstream and downstream of the farms, and 3 sites were from independent drainage systems not connected with either farm (Figure 1). Fish were briefly stunned with electrofishing equipment and netted into large holding tanks, quickly transported to the laboratory, kept in aerated tanks and dissected within 2 h of capture.

Fish were collected at the following sites (Figure 1):

a) Sites associated with Lag Vollagh Farm

Site 1 Corrany River: approx.100 m downstream of Lag Vollagh, Cornaa.

Site 2 Coranny River: approx. 100 m upstream of Lag Vollagh, Cornaa.

Site 3 Ballaglass Glen, Corrany River: approx. 500 m upstream of Lag Vollagh.

Site 4 Corrany River: approx. 2 km upstream of Lag Vollagh.

b) Sites associated with Riverside Farm, St John’s

Site 5 River Neb: approx. 100 m downstream of Riverside.

Site 6 River Neb: approx. 300 m upstream of Riverside.

c) Sites independent of water courses associated with both fish farms

Site 7 River Santon: section of river near village of Santon (south Isle of Man).

Site 8 Sulby River: Sulby Glen near village of Sulby (north Isle of Man).

Site 9 River Laxey: approx. 500 m upstream of the Laxey Wheel (north-east Isle of Man).

### Dissection protocol

Fish were killed with a sharp blow to the head immediately before examination in a field laboratory at the 2 farms. Weight, length, sex and haematocrit were recorded before dissection (data not considered here). The gill arches were transferred individually into 8 labelled Petri dishes in tap water and examined with a stereo microscope. Specimens of *D. sagittata* were counted, transferred singly to microscope slides and fixed flat in 10% formal saline under a glass coverslip for examination of the posterior haptor under higher (compound microscope) magnification.

### Determination of developmental stages and ages of *Discocotyle sagittata*

Each parasite was categorized into a developmental cohort, depending on the number of pairs of sclerotized haptoral clamps and the stage of reproductive development. Newly attached parasites have 1 pair of clamps and eyespots; the latter are subsequently lost before the 2nd pair of clamps begins to develop followed by 3rd and 4th pairs; after this, worms become sexually mature and begin producing eggs. This process of development has been measured in laboratory experiments at 13°C (Gannicott, 1997). The 2nd pair of clamps begins to develop after a minimum of 38 days, and further pairs are gained progressively until worms have 4 pairs of clamps after a minimum of 64 days. Worms become sexually mature after a minimum of 77 days. Timing of these events varies with seasonal environmental conditions; a schedule based on temperatures on the Isle of Man is cited in Tinsley *et al*. (2020). Estimates of age employing body length of fixed specimens is heavily dependent on fixation procedures; using pairs of sclerotized clamps as units of growth avoided these problems since these hard parts are unaffected by fixation protocols (Gannicott, 1997; Rubio-Godoy and Tinsley, 2002).

### Data analysis

Statistical analysis was carried out at the time of this study (Gannicott, 1997) using MINITAB. For the overdispersed frequency distributions of worm burdens, data were normalized with logarithmic transformations before analysis with General Linear Models. Post-hoc analysis (Tukey tests) examined differences between age cohorts and treatment groups in the farms. In a subset of the dissections of rainbow and brown trout at Lag Vollagh, infection data were recorded for fish distinguished as male, female or triploid. Age was not determined for fish in rivers but was unambiguous for farmed fish since these were reared as separate cohorts in age-specific ponds. Host sex was used as a covariate in comparisons of age cohorts in the farms. Age determination for parasites in farm and river populations employed haptor development in whole-mount microscope preparations of all individual worms (see above). For brown and sea trout in wild populations in rivers, comparisons of infection levels in different fieldwork locations were unrealistic: sample sizes were small, and a range of parameters allowing detailed analysis of infection levels were not recorded (e.g. host age, condition, and habitat characteristics including water flow rates).

Further analysis of the original data has not been possible because files have been lost in several ‘system upgrades’ to University storage over the years since the fieldwork. This accounts for the paucity of statistical detail (e.g. variation around the means for infection levels and the parameters of the GLM analysis, all recorded but then lost). The data shown here were included in a draft of this paper but this was put ‘on hold’ because the overall conclusion (no effect of farms on downstream fish populations) had no explanation. Only several years later, emerging information on the immune responses of trout to *D. sagittata* (see below) provided the present interpretation. So, whilst the statistical analysis based on MINITAB lacked the sophistication of current techniques employing ‘R’ (e.g. for *Discocotyle* in Tinsley *et al*. 2020), it nevertheless generated robust results.

## Results

### Levels of *Discocotyle sagittata* infection in the trout farms

During the study period for this account, 1993–1996, records of *D. sagittata* were compiled from Riverside and Lag Vollagh for a total of 447 rainbow trout and 109 brown trout. As a general guide to infections, combined over the 3 years, prevalence in rainbow trout at Riverside was 72% with mean intensity 59 worms/host (*n* = 255) and maximum burden 1150 worms. Prevalence in rainbow trout at Lag Vollagh was 91%, mean intensity 18·3 worms/host (*n* = 192), maximum 120. Infection levels in brown trout at Lag Vollagh (maintained in separate ponds from rainbow trout) were consistently lower: prevalence 64%, mean intensity 4.1 worms/host (*n* = 109), maximum 30.

**Figure 2.**
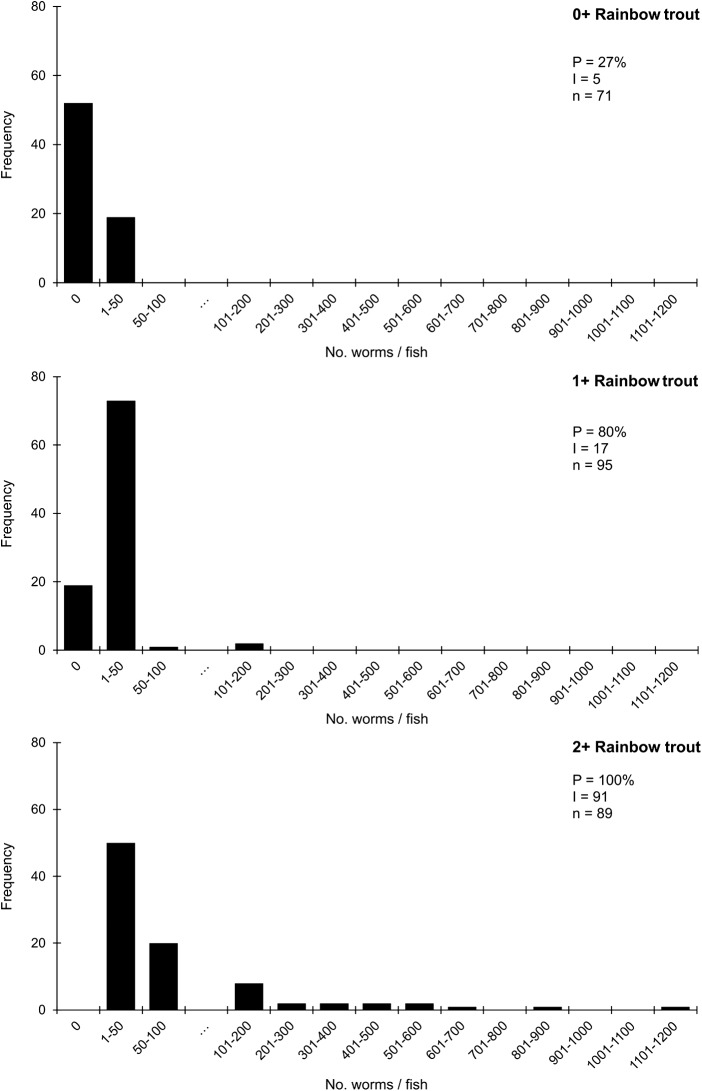
Frequency distributions of *Discocotyle sagittata* in 0+, 1+ and 2+ rainbow trout (*Oncorhynchus mykiss*) at Riverside farm, St. John’s. P = prevalence (%); I = mean intensity (worms/host); n = sample size. ‘…’ shows change of scale to provide more detail for the 1–100 burdens.

Variation in infection levels was associated with environmental conditions: elevated prevalence and intensity occurred in warmer drier summers attributable to increased rates of parasite egg production, faster development and maturation and to reduced water flows in farm ponds (data and analyses in Gannicott, 1997). Prevalence and intensity typically increased with host age (i.e. with duration of exposure for each cohort). For rainbow trout at Riverside Farm (Figure 2) prevalence increased from 27% amongst 0+ fish, to 80% for 1+, and 100% for 2+ fish, with mean intensity for these cohorts rising from 5 to 17 and 91 worms/host, respectively. Maximum burdens increased from 41 to 166 and 1150 for these years; the effect is shown by the frequency distributions in Figure 2 with a progressive extension to the right with a highly over-dispersed distribution and the disappearance of the uninfected class in year 2. The age cohort infections were significantly different from each other (GLM, ***P < 0·001) and the age-related increase was significant (post-hoc analysis, Tukey tests) (sample sizes in Figure 2).

**Figure 3.**
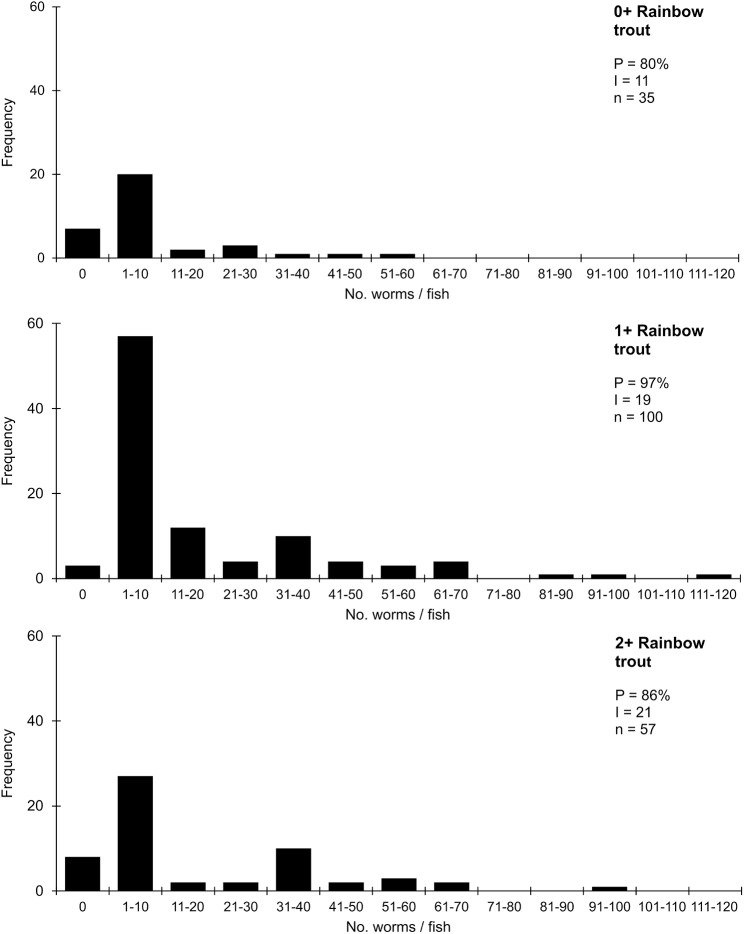
Frequency distributions of *Discocotyle sagittata* in 0+, 1+ and 2+ rainbow trout (*Oncorhynchus mykiss*) at Lag Vollagh farm, Cornaa. P = prevalence (%); I = mean intensity (worms/host); n = sample size.

For rainbow trout at Lag Vollagh (Figure 3), prevalence was high in all host age cohorts (80–97%); worm burdens were significantly different in the 0+ and 1+ samples (GLM, *P < 0.05, with post-hoc analysis, Tukey tests), with most attributes showing an approximate doubling between these year classes (mean intensity increasing from around 11 to 19 worms/host, maximum burdens 54 and 115, respectively). However, these measures, together with the histograms in Figure 3, show no continued change in the characteristics of the parasite populations between the 1+ and 2+ cohorts.

**Figure 4.**
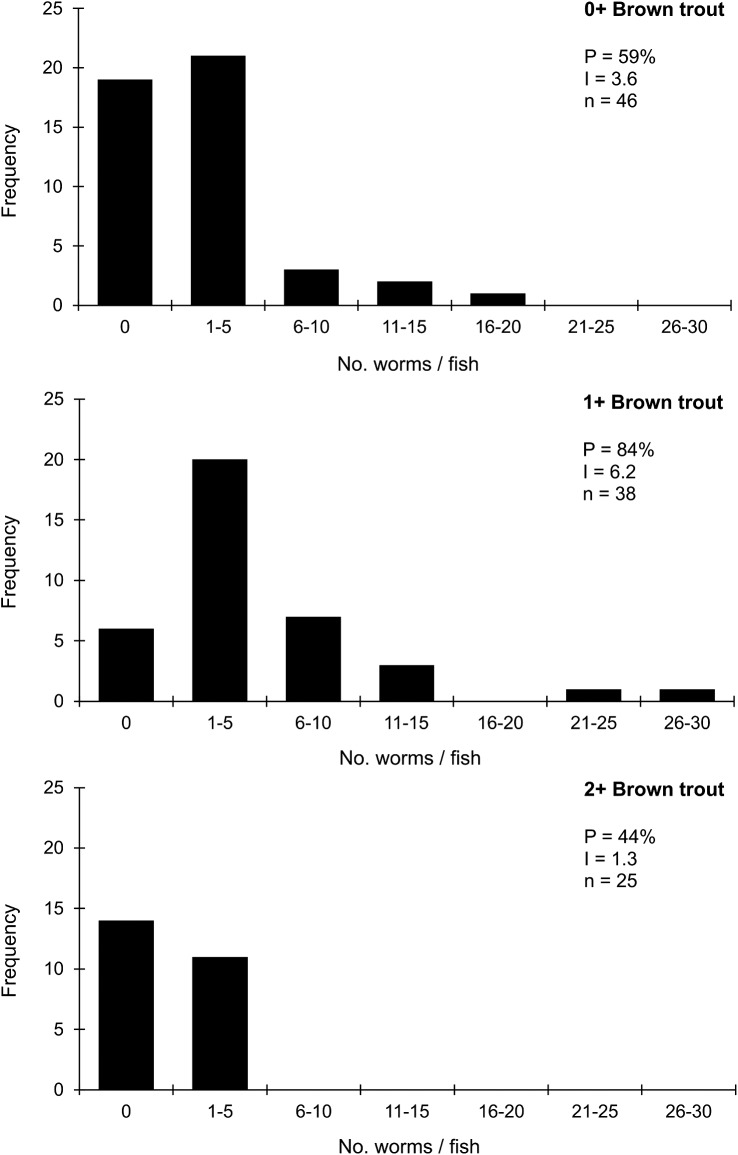
Frequency distributions of *Discocotyle sagittata* in 0+, 1+ and 2+ brown trout (*Salmo trutta*) at Lag Vollagh farm, Cornaa. P = prevalence (%); I = mean intensity (worms/host); n = sample size.

For brown trout at Lag Vollagh (Figure 4), with overall lower infection levels, age cohort comparisons showed increased prevalence between 0+ and 1+ fish and an approximate doubling of mean intensities (from 3.6 in 0+ fish to 6.2 in 1+ fish) and maximum burdens (20 and 30, respectively). However, there was a precipitous decrease in all infection attributes in 2+ brown trout (prevalence 44%, mean intensity 1.3 worms/host, and no fish carried more than 5 worms: exceptionally, more fish in the samples were uninfected than infected (Figure 4). Worm burdens were significantly different in the 3 age cohorts (GLM, ***P < 0.001) and post-hoc Tukey tests demonstrated that burdens were significantly higher in 1 year old brown trout compared with either of the other groups.

Comparison of worm burdens in male, female and triploid rainbow trout in 1+ and 2+ age classes showed no significant differences (overall *n* = 341 fish). For the lower burdens in brown trout, there was again no significant influence of host sex on intensities of infection in either of the 2 age classes (GLM, *P > 0.05 in all comparisons, data not considered further).

### Infections of *D. sagittata* on sea trout

**Figure 5.**
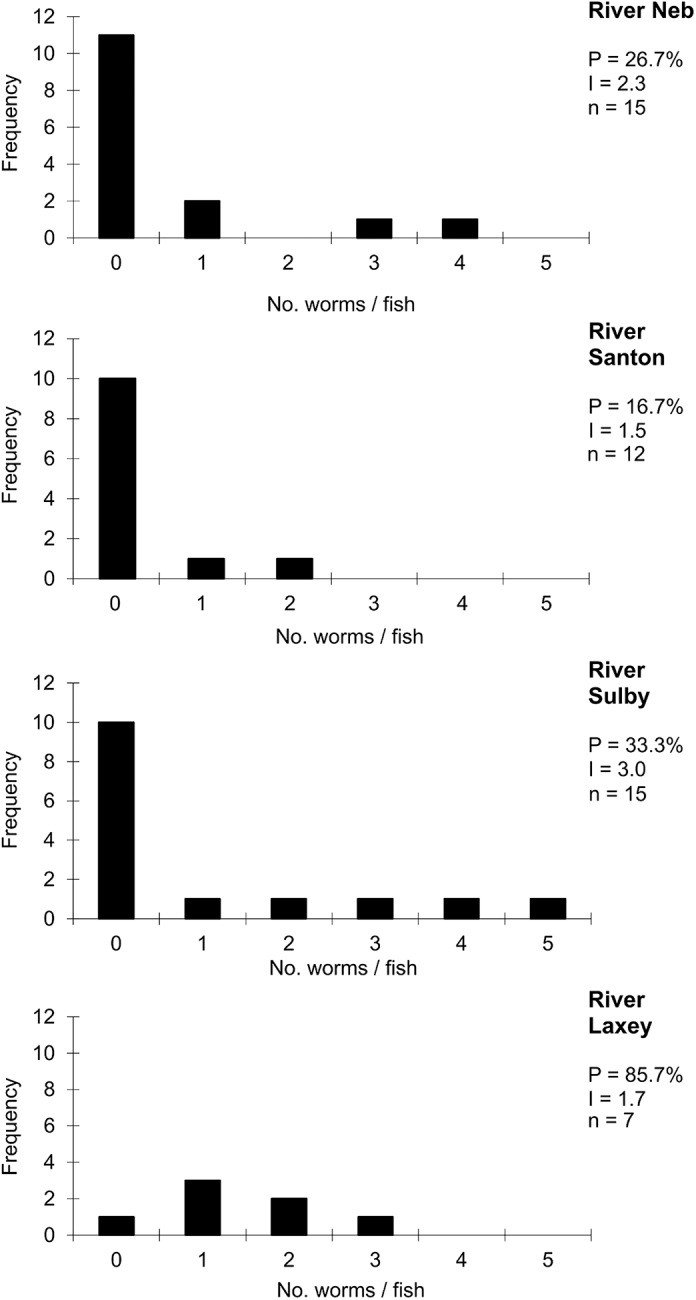
Frequency distributions of *Discocotyle sagittata* in sea trout (*Salmo trutta*) in rivers on independent drainage systems. P = prevalence (%); I = mean intensity (worms/host); n = sample size.

**Figure 6.**
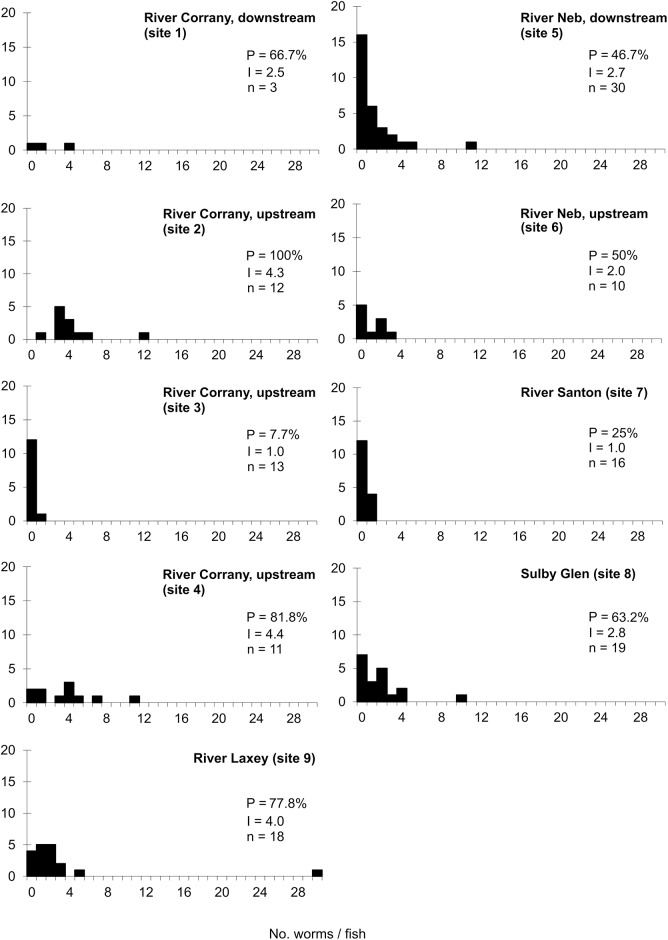
Frequency distributions of *Discocotyle sagittata* in brown trout (*Salmo trutta*) in rivers on independent drainage systems. P = prevalence (%); I = mean intensity (worms/host); n = sample size.

Sea trout were collected in 4 of the 5 watercourses sampled (Figure 1); *D. sagittata* occurred in all 4, alongside infections in brown trout (compare Figures 5 and 6). Frequency distributions of parasites infecting sea trout in the Rivers Neb, Santon, Laxey and Sulby are shown in Figure 5 with maximum burden 5 worms/host (at Sulby). Prevalence at each site ranged from 17% (River Santon) to 86% (River Laxey), with mean intensities within the narrow range 1.5 to 3.0 worms/host (*n* = 49 fish). Mean intensity for the sample associated with a farm (Riverside Farm, River Neb, 2.3 worms/host, *n* = 15 fish) cannot be distinguished from the worm burdens in the 3 independent rivers (1.5, 1.7, and 3.0 worms/host, *n* = 12, 7, and 15 fish, respectively; Figure 5). Age analysis of *D. sagittata* across the overall sample of sea trout indicated 2 distinct developmental cohorts: early adult stages just pre-egg laying and recently attached juveniles with 1 pair of haptoral clamps.

### Infections of *D. sagittata* on wild brown trout

Prevalence and mean intensity of infection in brown trout at 9 sites are listed in Figure 6. Prevalence ranged from 8% (site 3) to 100% (site 2) with mean intensity 1.0 and 4.3 worms/host, respectively. These extremes of variation were recorded on the same river approximately 400 m apart. Frequency distributions of parasite burdens at each site (Figure 6) show the highest intensity was 30 worms/host found in the River Laxey (site 9), but all other fish from this site carried ≤5 worms/host. Maximum intensities at the other 8 sites were all ≤12 worms/host, with 95% ≤5 worms/host. Mean worm burdens in wild brown trout from across this survey fell within a narrow range: 1–4 worms/host (Figure 6).

Infection levels in *D. sagittata* in brown trout from rivers associated with the farms and in those from independent drainages were closely comparable. In the Rivers Corrany and Neb, local to the farms, 54% of fish were infected with an average of 3.4 worms/host (*n* = 79), whereas 57% of fish from separate watercourses (Rivers Laxey, Santon and Sulby) had mean intensity 3.1 worms/host (*n* = 53).

## Discussion

The survey of trout in 5 widely separated watercourses shows that *D. sagittata* is endemic in the Isle of Man, infecting brown trout, sea trout and rainbow trout. These rivers are independent of each other with outlets into the sea on the North, Northeast, South and Southwest coasts of the island. Parasite burdens in natural watercourses were low (mean intensities typically ≤5 worms/host) but heavy infections occurred in 2 farms rearing trout, principally rainbow trout, with numbers of worms in 2+ rainbow trout commonly exceeding 100, up to 1150/host.

The high levels of farm infection, associated with significant pathology and mass mortality in some years, recurred during long-term records beginning in the 1980s (Ronga, 1993) and documented by 16 further references until concluding fieldwork in 2012 (Tinsley, *et al*. 2020). It would be entirely predictable,therefore, to expect free-living fish in rivers receiving drainage from the trout farms to be exposed to considerably greater infection risk than under natural circumstances and that this would be reflected in elevated parasite populations. However, the present survey has recorded levels of infection downstream of the farms that cannot be distinguished from those in independent rivers.

This fieldwork has provided a guide to the characteristics of infection with *D. sagittata* on the Isle of Man, the roles of the different hosts in maintaining it, and the evidence for assessing the impact of farm infections on disease risks in wild trout populations.

## Discocotyle sagittata *in the fish farms*

Factors contributing to the heavy infections of farmed trout relate particularly to the organization and management of the 2 farms. At both, new fry were initially maintained in separate raceways without contact with older, infected fish; they developed their first infections from parasite eggs and larvae washing into the farm system from wild fish in the adjacent river. In the autumn of their first year, these fish were transferred into the first of a series of ponds just vacated by older infected fish. Crucially, the latter left behind eggs in the sediment which hatched and generated the first increment to infection of the incoming 0+ fish (Gannicott, 1997). In the following years, prevalence and intensity increased with successive exposures. Heavy infections were self-reinforcing: high mature worm burdens had high egg output leading to higher invasion rates. Ponds at Riverside had mud substrates trapping a greater proportion of accumulated eggs. With limited water catchments upstream of the farms, reduced water flows in summer kept accumulated eggs within the pond populations. In dry summers, water flows were maintained by pumping water from ponds at the lower end of the farm up to the topmost ponds: this recirculated eggs and oncomiracidia through the series of ponds which would otherwise be lost in the outflows to the river. As in most farming systems, fish were confined at high density increasing stress with, amongst other effects, possible negative impacts on host immune responses.

The presence of farms with heavy infections of *D. sagittata* along the course of waterways has the potential for 2 effects on the associated native trout populations. First, large numbers of parasite eggs and hatched infective stages are likely to increase infection pressure on wild trout. Second, trout escaping from farms may carry their infection into adjacent rivers, releasing eggs directly into habitats occupied by wild fish. The former risk of infection is likely to be substantial: in summer, at temperatures around 18°C, adult worms produce a mean of *c.* 12 e/w/d (Gannicott and Tinsley, 1998a); with average burdens exceeding 50 worms/host, 1000 fish would produce over 0 6 million e/d. Lag Vollagh farm had 10 ponds each typically containing 5–6000 fish. It is not known what relative proportions of egg output accumulate in the pond sediment compared with those redistributed into the water column and then washed out of the farm. However, given the large fish populations and heavy infection levels, even a small proportion of eggs passing downstream could pose a significant risk of infection to fish below the farm outlet. Larvae survive for over 24 h at average summer temperatures, increasing the downstream dispersal with water currents (Gannicott and Tinsley, 1998a,*b*). The additional disease risk, from escape of infected farm fish into natural trout populations, creates the possibility of much wider and longer-term parasite dispersal including, importantly, the spread of infection upstream of the farm source.

## Discocotyle sagittata *in rivers: sea trout*

The age structure of parasite populations on the Isle of Man shows that environmental conditions typically allow continuous transmission throughout the months when water temperatures are ≥ 10 °C (data in Gannicott, 1997; Rubio-Godoy and Tinsley, 2008a). Worms in each developmental stage (i.e. with increasing numbers of haptoral clamps) typically form a continuous series representing the month-by-month succession of invasions (see also Tinsley *et al*. 2020).

The age structure of *D. sagittata* on sea trout does not show a similar continuous invasion history, reflecting instead interruptions created by migrations between marine and freshwater environments. The parasite survives on fish in seawater (Slinn, 1963, and present data), but eggs fail to develop in brackish and seawater, so transmission is limited to the period in freshwater (Gannicott, 1997). The rates of development of eggs and post-infection stages are temperature-dependent and, in most years, transmission is largely inhibited between late November and early May (Gannicott, 1997; Tinsley *et al*. 2020). This contributes to a relatively narrow window for sea trout to acquire infection, beginning as they migrate into rivers. The developmental stages of parasites in present samples indicated 2 age classes: adults (with 4 pairs of clamps) and recently attached juveniles (1 pair of clamps). The adults were likely to have invaded in one or more previous years, surviving at sea between excursions into freshwater: these could not have developed from invasion to maturity in the short time the fish had been in freshwater. The juvenile worms had probably invaded during the few weeks before the fish were caught, after migration to freshwater, but their further development (after this sampling date in November) would be arrested over winter and they would not reach maturity until the following summer (see schedule in Tinsley *et al*. 2020). The source of these new infections is likely to have been brown trout (with overlaps in data for habitats and sampling dates). At the time of this study, the brown and sea trout had similar mean intensities of infection where they co-occurred: 1.5, 1.7, 2.3 and 3.0 for sea trout (Figure 5) and, for wild brown trout at the same 4 sites, 1.0, 4.0, 2.4 and 2.8 worms/host (Figure 6). This ‘snap-shot’ of infections in sea trout and brown trout suggests little difference in their contributions to transmission from the egg output of the adult worm burdens in summer, but the duration of the contribution from sea trout is limited by their migration back to sea. Nevertheless, sea trout can potentially have a significant role in the spread of infection when they carry established parasites that have survived at sea into freshwater outlets populated by uninfected brown trout.

The sea trout studied by Slinn (1963) off the coast of the Isle of Man had prevalence of infection *c.* 24% (*n* = 162 fish), with intensities 1 to 14 (mean 4.2) worms/host. In the present samples of sea trout in freshwater, mean intensities per site were generally lower than recorded by Slinn (1963) and no burden was greater than 5 worms/fish (Figure 5).

## Discocotyle sagittata *in rivers: Between-population comparisons in free-living brown trout*

The occurrence of *D. sagittata* on wild brown trout in all the rivers studied indicates that parasite infections are self-maintaining, without any role of farmed fish in their persistence. The major focus for this study has been to detect elevated parasite populations in wild fish that might reflect the massive increase in egg numbers downstream of the farms. In particular, between-population comparisons have looked for ‘hotspots’ where pathogenic effects might constitute parasite-induced disease.

Considering the highest intensities at each site that might indicate potential ‘hotspots’, there were 5 locations with individual burdens of ≥10 worms/host: 2 were upstream of a farm, 1 was downstream and 2 were in independent rivers. The highest worm burden across the overall sample of 132 free-living brown trout was 30 parasites at Laxey, a river without connection to the nearest farm at Lag Vollagh. Environmental influences on transmission are unknown but the next largest burden in this host sample was 5 while the other infected fish carried only 1–3 worms (Figure 6). All other records for individual fish had maximum burdens of ≤12 worms. Considering the burdens potentially associated with loss of condition in rainbow trout (perhaps around 100 worms/host, see below), there was nothing to indicate potential ‘hot-spots’ of high intensity that might constitute a pathogenic worm burden in brown trout; indeed, infection levels in wild brown trout associated with the 2 farms were almost identical to those in independent drainages: prevalence 54% and 57%, mean intensity 3.4 and 3.1 worms/host, respectively.

At a wider scale, the present records of infection levels in the wild brown trout on the Isle of Man are comparable with those found elsewhere in the United Kingdom (see Thomas, 1964; Chubb, 1977). These studies have noted variable prevalences but, across an overall sample size exceeding 750 fish, mean intensity in almost all the individual populations studied did not exceed 6 worms/host (Gannicott, 1997). The single exception to this limit was a record of 8 worms based on a sample of 2 fish (Friend, 1939) (still far below any association with disease). Other constituent samples in these studies (each based on *n* = 56–334 fish) recorded mean intensity 5.7 (Chubb, 1977), 4.4 (Llewellyn and Owen, 1960), 0.5–1.2 (Thomas, 1964), 0.3–3.5 (Paling, 1965), 2.0 (Wootten, 1973) and 3.3–4.8 worms/host (Kennedy *et al*. 1991). These populations represented contrasting environmental conditions in Wales, Scotland and England but provide an indication of very limited variation. In the present study, mean intensities in population samples of brown trout fell within a similar range with an average across all rivers of *c.* 3 worms/host (Figure 6).

The extremes of variation in prevalence across the 5 rivers sampled (8% and 100%) were recorded on the same day in the same river upstream of Lag Vollagh farm, at sites only 400 m apart, based on similar sample sizes (13 and 12). This highlights the difficulties in assessing variation and in comparing infection levels in different populations. Prevalence provides a relatively insensitive comparison of infection levels (e.g. Jovani and Tella, 2006), further limited in this study by sample size. Most importantly, the *Discocotyle/*rainbow trout interaction has been shown to be characterized by considerable variation in infection rates at an individual host level. Tinsley *et al*. (2020) exploited exceptional circumstances at one of the present farms (Riverside) where a population of rainbow trout had been maintained undisturbed in a single pond, exposed to year-on-year re-infection from its self-contained *D. sagittata* infection. Worm burdens at the time of sampling represented the endpoint of infection events accumulating over 9 years for hosts of the same age, sharing the same habitat and environmental conditions. Prevalence was 100% but worm burdens in a sample of 20 fish varied from 10 to 2628 worms/host, with mean intensity 576 worms/host (on rainbows much larger than typical in farm rearing). Within the sample of 20 fish, the infrapopulations were highly heterogeneous: the 4 fish (i.e. 20%) with the highest burdens carried 58% of the total parasite population (6666 out of the total of 11526 worms, with each of these 4 hosts carrying > 1000 worms); the same proportion of fish with the lowest intensities (10–58 worms/host) carried only 1% of the total parasite population (Tinsley *et al*. 2020).

With such wide heterogeneity it is unrealistic to attempt detailed statistical analysis of infection levels based on small sample sizes. But, whatever between-population differences may occur, the main focus of this study looks for evidence of pathogenic infection levels attributable to disease over-spill from farms (the ‘hotspots’). Here, the lack of such evidence is conclusive. With reference to burden sizes that might constitute parasite-induced disease, studies on farmed rainbow populations and laboratory infections have shown a significant negative correlation between parasite intensity and host haematocrit in burdens of *c*. 100 worms/host (Gannicott, 1997). In addition, Rubio-Godoy and Tinsley (2004b, 2008a) recorded that intensity is negatively correlated with the coefficient of body condition (fish weight and length). In this later study, the capacity for extreme pathological effects was illustrated by 1 year class of 2+ rainbows at Riverside where prevalence was 100% with intensities of 60–255 (mean 160) worms/host. High worm burdens were accompanied by gill tissue damage, severe anaemia, respiratory distress and mass mortality; surviving fish were culled because of their poor condition, resulting in the loss of the entire year class of thousands of fish (Rubio-Godoy and Tinsley, 2008a). While fine-scale effects on fish health are undetermined, the overall mean intensity in farmed rainbow trout in the present study exceeded 50 worms/host (data for both farms and all age classes and years combined). The overall mean intensities on wild brown trout were characteristically around 5 worms/host and their blood feeding is unlikely to represent a significant cause of disease.

It remains difficult to reconcile the mismatch between theoretical expectations of elevated infection levels downstream of the farms and the observed results indicating no detectable effect on wild populations. It is possible that the change in transmission environment contributes. Despite the massive outflow of parasite eggs and infective larvae with farm effluent, infectivity might be negatively affected by the transfer from relatively static, close host–parasite contact in farm ponds to the dynamic conditions of flowing river water. However, physical and chemical changes accompanying discharge into the riverine environment are unlikely to explain the absence of hotspots of infection. Alternatively, rather than an external environmental effect influencing the outcome of invasion from increased infection pressure, the internal host environment may be responsible, reflecting immune ability to control parasite survival post-infection.

### Parasite–host interactions: evidence for immune control of worm burdens

Immunological interactions have been characterized in the trout – *D. sagittata* relationship, demonstrating that both innate and acquired host defences partially regulate parasite burdens. In controlled experimental infections, rainbow trout were shown to be more susceptible to infection than brown trout (Rubio-Godoy and Tinsley, 2004a): this may be partially explained by the finding that complement of the native trout is more effective at killing *D. sagittata* oncomiracidia than that of the introduced salmonid (Rubio-Godoy *et al*. 2004). Acquired immunity is also involved in controlling *D. sagittata*: vaccination of *O. mykiss* with parasite extracts elicits production of specific antibodies which provide partial immunity to infection (Rubio-Godoy *et al*. 2003b). In laboratory experiments, rainbow trout exposed to repeated trickle infection developed significant partial protection against subsequent challenge: reduced worm burdens were recorded 5 months after the start of the infection regime, although no correlation was observed between infection and antibody levels (Rubio-Godoy and Tinsley, 2004b). Similarly, in fish farms, both rainbow trout and brown trout produced antibodies against *D. sagittata* but, again, no association was found between infection levels and antibody titres (Rubio-Godoy *et al*. 2003a). Thus, trout can develop immunity against *D. sagittata* involving both specific and non-specific components: these most likely act in combination, hence the difficulty in correlating protection with a particular immune component. Gilthead seabream *Sparus auratus* develop acquired immunity against the gill-infecting monogenean *Sparicotyle chrysophrii* with protection mediated by parasite-specific immunoglobulin secreted via the gill mucosa (Riera-Ferrer *et al*. 2025).

The previous studies at Riverside farm, described by Tinsley *et al*. (2020) and above, revealing wide heterogeneity in intensities (from tens to thousands of worms/host), demonstrated significant individual-level control of burdens where fish showed a consistent response to invasion relative to one another. On the basis of haptor development (and hence age) the overall parasite population could be sub-divided developmentally and chronologically into cohorts (adults, sub-adults, juveniles, post-larvae) and the burden sizes acquired during each corresponding time period were highly consistent for each host (Tinsley *et al*. 2020). The rank order of burden sizes showed close concordance across the host population sample, indicating a highly structured parasite suprapopulation. Tinsley *et al*. (2020) concluded that level of infection was a repeatable characteristic of individual hosts with statistically-significant co-variance between intensities of successive developmental cohorts on each fish (i.e. successive periods of invasion). Fish characterized by low parasite burdens following each successive challenge may be considered relatively resistant, expressing an inherent ability to limit infection; fish with high intensities of each age cohort demonstrated a weaker ability to control parasite survival.

These data relate to farmed rainbow trout, but field and laboratory studies (above) indicate that brown trout have greater immunity to *D. sagittata* than rainbow trout. So, taken together, these findings support the conclusion that the absence of an expected increase in infection levels in brown trout downstream of farms may be attributed to immune control of parasite survival post-infection. A further measure of the relative effectiveness of immune control in brown and rainbow trout is provided by observations at Lag Vollagh farm where both species are raised (in separate but contiguous ponds). Data amalgamated across the 3 years 1993–1996 recorded prevalence in rainbow trout 90%, mean intensity 18.3 worms/host compared with prevalence in brown trout 64%, mean intensity 4.1 worms/host (see above and Gannicott, 1997). The comparison may only be indicative rather than precisely matched: the overall data were not controlled for differences between constituent samples in host age classes, pond of origin, parasite developmental stages (i.e. duration on the host), time of year and temperature, all of which would influence infection levels at the time of sampling. Nevertheless, the broad comparison supports more precise data that brown trout characteristically carry lower infection levels than rainbow trout.

Direct visualization of the relative effectiveness of immune control in brown trout was provided by the comprehensive monitoring of infections at Lag Vollagh farm by Gannicott, (1997). Data described above and in Figures 3 and 4 followed 3 successive annual transmission seasons for rainbow and brown trout. Typically, infection levels increased for both hosts between years 1 and 2 (reflecting additional exposure) while a negligible change for rainbow trout in year 3 was suggestive of increasing partial protection. However, in brown trout, levels of infection showed a very considerable reduction in year 3, significantly different even from those acquired in the first year, suggestive of much more effective regulation than in rainbow trout. Frequency distributions for these brown trout (Figure 4) confirm this downward trajectory in 2+ fish with prevalence about half and intensity only about one-fifth of those in the previous year class despite the further year’s exposure (data above and Gannicott, 1997). The direct relevance of this example concerns the arrangement of the ponds and their sources of water. The brown trout pond was directly below that of the heavily infected rainbow trout (Figure 3) and received its main water inflow (and parasite eggs and oncomiracidia) from above.

The decline in infection levels in older brown trout was recorded again at the same farm in 1999–2001 (Rubio-Godoy and Tinsley, 2008a). In 1+ and 2+ brown trout, mean intensities were <5 and 3 worms/host (maxima 15 and 4), respectively, contrasting with infections in the equivalent age classes of rainbow trout in adjacent ponds with mean intensities of 48 and 132 worms/host (maxima 92 and 311), respectively, for 1+ and 2+ fish. Again, the brown trout population received its main water supply from ponds higher in the farm system containing infected rainbow trout.

These circumstances represent a close analogy to the question central to the overall remit of this study – to determine whether egg output from heavily-infected farms increases infections in downstream river fish. Indeed, the test of this hypothesis within the farm was even more rigorous than that experienced by brown trout in the river environment: exposure in the farm represented a more immediate delivery of infection with both host species in the same environmental conditions. Significantly, despite this direct challenge from adjacent rainbow trout, the 3 years of accumulated transmission for the brown trout in both data sets produced final infection levels far below those constituting a disease risk – even within Lag Vollagh farm where brown trout faced intense attack ‘inside the lion’s den’.

The presumed immune regulation recorded in this study does not result in complete parasite population elimination, although prevalence may fall. Despite huge invasion challenge, *D. sagittata* populations in the farmed brown trout may persist at intensities closely comparable with those independent of fish farm drainage on the Isle of Man and, more widely, in literature records over the range of *D. sagittata* (references above). Thus, the infections in brown trout are characteristic of their host–parasite relationship (mediated by innate and acquired immunity) rather than an effect of the force of infection in a specific environment.

## Conclusions

The outcome of this study addresses 2 questions. First, what is the extent to which the spillover of parasite infective stages from heavily infected fish farms increases parasite burdens and hence disease risks in downstream populations of wild fish? Results show conclusively that this does not occur in this host–parasite system. Indeed, from these data, any threat of significant disease can be excluded entirely: the severe pathology (and mortality) recorded in the farms is associated with burdens approaching and exceeding 100 worms/host; very few fish in the rivers carried more than 10 worms and most intensities were <5 per host. The second question follows from the first: why is the expected negative impact of infection pressure not encountered? Here there is strong evidence that, regardless of any impact of elevated numbers of infective stages in the environment, the parasite populations that establish post-infection are regulated by powerful host immunity, reducing the surviving parasite infrapopulations to levels characteristic of the *S. trutta–D. sagittata* interaction. This case study has wider application in ecological parasitology. It adds to other investigations of monogenean with lower vertebrate (i.e. ectothermic) associations providing empirical measures of the extent of immune-mediated parasite attrition. In contrasting circumstances in amphibian hosts – one fully aquatic and the other adapted to deserts – there is field and laboratory evidence that successful initial infections of *Protopolystoma xenopodis* in *Xenopus laevis* and *Pseudodiplorchis americanus* in *Scaphiopus couchii* are reduced post-infection so that around 95% of worms invading successfully are eliminated before maturity (Tinsley, 1999; Tinsley and Jackson, 2002; Tinsley *et al*. 2012). This attrition represents a major constraint on factors including pathology, reproductive output and future transmission which have fundamental implications for host–parasite relationships.

These findings are relevant to aquaculture and to the general threats to native fauna from farmed animal species. However, the implications do not establish a general principle relating to spillover of disease from fish farms: the outcome is specific to this host species/monogenean interaction based on the relative resistance of *O. mykiss* and *S. trutta* to the same parasite and does not apply to potential disease spillover (or spillback) with other pathogens. From an evolutionary perspective, the differing responses of rainbow and brown trout to *D. sagittata* may reflect the differences in the association of these 2 host species with the single parasite species. *Discocotyle sagittata* is a native infection of brown trout in the United Kingdom with rainbow trout a relatively novel host, introduced only in the 1880s. The very variable defence in rainbow trout is illustrated by the record of 9 year’s exposure (above and Tinsley *et al*. 2020) where some fish carried a few tens of worms/host while others, alongside, carried > 1000 worms/host. This contrasts with a perhaps more stable relationship between brown trout and *D. sagittata* where most burdens are < 10 worms/host.

The present empirical data have wider relevance in Parasitology concerning individual-level variation in the host response to infection. The wide variation in infection levels in rainbow trout mirrors that indicated by molecular analysis of responses to immune challenge in wild rodents by Wanelik *et al*. (2020). Their transcriptome-wide analysis at the level of genes demonstrates categories of variability in response to infection; the consistent within-population heterogeneity in *D. sagittata*, characteristic at the level of individual hosts, provides empirical documentation of responses in wild fish infections. This monogenean system shows that infection levels in the natural host species, brown trout, may be maintained within narrow limits despite variation in environmental factors that might determine invasion success. Individual parasite burdens – and hence disease – may be influenced relatively less by infection pressure operating pre- and during invasion and more by host-immunity exerted post-infection.

## Data Availability

All original data are contained in the thesis by Gannicott (1997).
